# LINC00839 in Human Disorders: Insights into its Regulatory Roles and Clinical Impact, with a Special Focus on Cancer

**DOI:** 10.7150/jca.93820

**Published:** 2024-02-25

**Authors:** Yingqiu Hu, Yushan Hu, Xuan Lu, Hongliang Luo, Ziwen Chen

**Affiliations:** 1 Emergency Department, The Second Affiliated Hospital, Jiangxi Medical College, Nanchang University, Nanchang 330008, Jiangxi, China; 2 Department of Gastrointestinal Surgery, Ganzhou Hospital Affiliated to Nanchang University, Ganzhou, 341000, Jiangxi, China; 3 Second School of Clinical Medicine, Jiangxi Medical College, Nanchang University, Nanchang 330008, Jiangxi, China; 4 Department of Gastrointestinal Surgery, The Second Affiliated Hospital, Jiangxi Medical College, Nanchang University, Nanchang 330008, Jiangxi, China.

**Keywords:** LINC00839, Neoplasms, Non-neoplastic, Biological Functions, Regulatory Mechanisms, Clinical application

## Abstract

LINC00839 has captured significant attention within a spectrum of human disorders, including acute lung injury, osteoarthritis, and childhood obesity. Notably, aberrant expression patterns of LINC00839 have been observed across diverse cancer tissues and cell lines. LINC00839 emerges as an oncogenic factor in tumorigenesis and exerts a positive influence on tumor-associated behaviors. Its therapeutic potential for various cancers is underscored by its modulatory impact on pivotal signaling pathways, such as PI3K/AKT, OXPHOS, and Wnt/β-catenin. Additionally, LINC00839's role in reducing sensitivity to drug and radiotherapy interventions presents opportunities for targeted intervention. Furthermore, elevated LINC00839 expression indicates advanced clinicopathological features and foretells unfavorable prognoses, as validated by publications and comprehensive analyses of tumor types using TCGA datasets. This review elucidates the multiple regulatory mechanisms and functional implications of LINC00839 in various diseases, especially malignancies, emphasizing its potential as a predictive biomarker and therapeutic target across multiple disease domains in humans.

## Introduction

Long non-coding RNAs (lncRNAs) constitute a subset of non-coding functional RNAs that span over 200 nucleotides 1, 2. In recent years, with rapid advancements in RNA sequencing and array technologies, a plethora of non-coding RNAs, including lncRNAs, have been unearthed 3-6. The identification of novel forms of lncRNAs has piqued the interest of researchers, driving them to elucidate their biological roles. Accumulating evidence has highlighted the connection between the dysregulation of lncRNAs and the initiation and progression of diverse disorders, such as cardiovascular disease 7-9, cholestatic liver disease 10-12, and particularly malignancy 13-16. Furthermore, in-depth investigations have illuminated the pivotal role that lncRNAs play in the development of various diseases, exerting influence over processes such as lipid metabolism 17, 18, cellular aging 19-22, and an array of biological behaviors associated with tumors 23-28, including cell migration, invasion, and metastasis. This paradigm extends further as lncRNAs have risen as promising candidates for clinical applications, owing to the deepening exploration of the molecular mechanisms that govern the multifaceted functionalities of numerous lncRNAs.

Homo sapiens (human) long Intergenic Non-Protein Coding RNA 839 (LINC00839) isan RNA gene classified as lncRNA. With the location of 10q11.21, this gene has 5 exons and spans a length of 19,847 nucleotides(nt) (https://www.ncbi.nlm.nih.gov/gene/84856). The lncRNA transcripts produced by this gene exhibit nine distinct splice variants, each with varying sizes, ranging from 1845 base pairs (bp) for LINC00839-206 to3982 bp for LINC00839-204 (https://www.ensembl.org/Homo_sapiens/Gene/Summary?g=ENSG00000185904;r=10:42475480-42495337).

**Figure 1:**
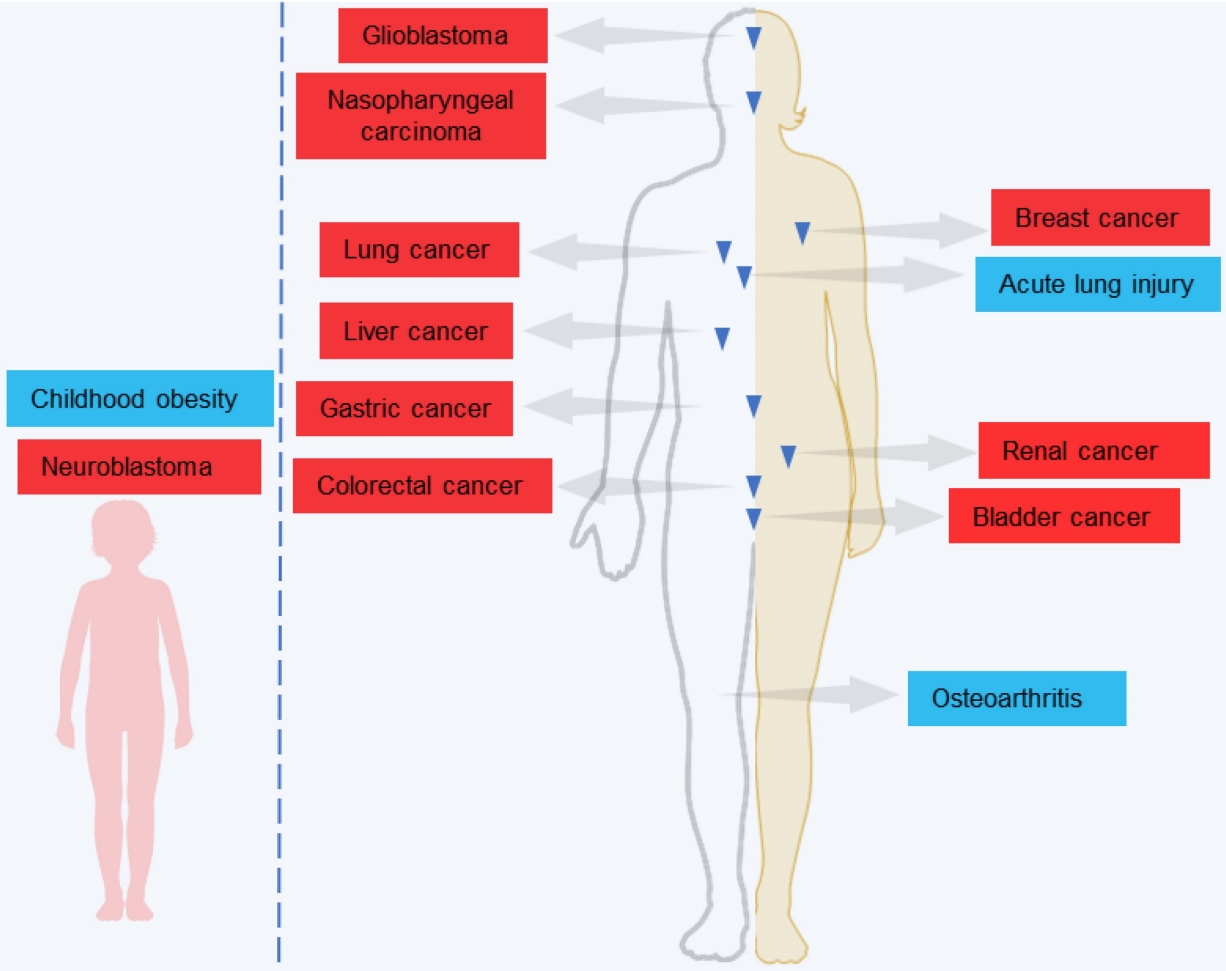
LINC00839 has been studied in a variety of human non-neoplastic and neoplastic diseases in children and adults.

LINC00839 has been implicated in a variety of human diseases and pathophysiological processes (**Figure 1**). For non-neoplastic diseases, LINC00839 plays a pivotal role in the pathogenesis of acute lung injury 29, osteoarthritis 30, and childhood obesity 31. Remarkably, its involvement has garnered growing interest in tumors. LINC00839 exhibits differential expression and may play important roles in a series of human tumors 32-47, including nasopharyngeal, breast and lung cancers. High expression levels of LINC00839 in tumor tissues indicated poor clinical outcomes with unfavorable clinicopathological features and prognosis in tumor patients, including lymph node metastasis, clinical stage, and overall and disease-free survival. Moreover, LINC00839 regulated tumor progression and development by mediating multiple vital biological processes, and tumor-related signaling. Considering its pivotal role in disease pathogenesis and progression, LINC00839 is anticipated to serve as a valuable biomarker and effective therapeutic strategy in diverse types of diseases.

In this review, we present a comprehensive summary of the most recent research progress elucidating the roles played by LINC00839 in the development of human neoplastic and non-neoplastic diseases. We focus on LINC00839 potential as a promising disease biomarker, as well as its biological functions in disease development. In addition, we delved into the underlying mechanism of LINC00839's action and explored its potential clinical application in multiple disease settings and analyzed the relationship between LINC00839 and the prognosis of more tumors using the Cancer Genome Atlas (TCGA) dataset. This review sheds light on the promising prospects of LINC00839 as a target for therapeutic interventions in various disease types.

## Non‑cancerous diseases

Research on LINC00839's involvement in non-cancerous diseases remains limited, including acute lung injury 29, osteoarthritis 30, and childhood obesity 31 thus far. These studies mainly unveiled the function of LINC00839 through predictive analyses employing total RNA-seq or assessments utilizing online datasets. Further experimentation is needed to substantiate these findings and delve deeper into its mechanisms.

### Acute lung injury

Acute lung injury (ALI) stands as a grave complication arising from various diseases, exhibiting a notable prevalence and mortality rate within clinical settings 48, 49. In a study by Fu et al. 29, it was demonstrated through experimental investigation that LINC00839 has the capability to function as a competing endogenous RNA (ceRNA) against miR-223. This action subsequently leads to an elevation in NLRP3 levels, thereby fostering inflammation and pyroptosis induced by LPS. Interestingly, the application of sevoflurane, a recognized protective agent for ALI affecting the lungs, was able to counteract these effects. Given these findings, LINC00839 emerges as a promising and novel candidate worthy of consideration for targeted gene manipulation in the treatment of ALI.

### Osteoarthritis

Osteoarthritis (OA) represents a degenerative joint ailment linked to age, exhibiting a growing prevalence worldwide and a dearth of impactful remedies 50, 51. In the research by Chen et al. 30, it was found that LINC00839 showcased significant upregulation in osteoarthritic cases in contrast to normal knee samples. Additionally, a central network of long non-coding RNAs (including LINC00839) was established, potentially involved in osteoclast differentiation and the orchestration of extracellular matrix structure during osteoarthritis progression. Although these findings offer valuable insights, further experimental investigations are imperative to validate these associations.

### Childhood obesity

Childhood obesity is one of the most serious health problems worldwide 52, with its roots often tracing back to shifts in lifestyle and dietary patterns during early years 53, 54. Within the context of childhood obesity, an intriguing finding emerges from a study where LINC00839 exhibited significantly elevated expression levels in obesity-afflicted samples compared to their normal counterparts 31, and LINC00839 has been identified as a pivotal RNA participant, engaged in the MAPK signaling pathway and apoptosis 31. Thus, LINC00839 might be one key regulator involved in childhood obesity 31, that might provide useful information for exploring new biomarkers and therapeutic targets.

## Cancerous diseases

**Figure 2:**
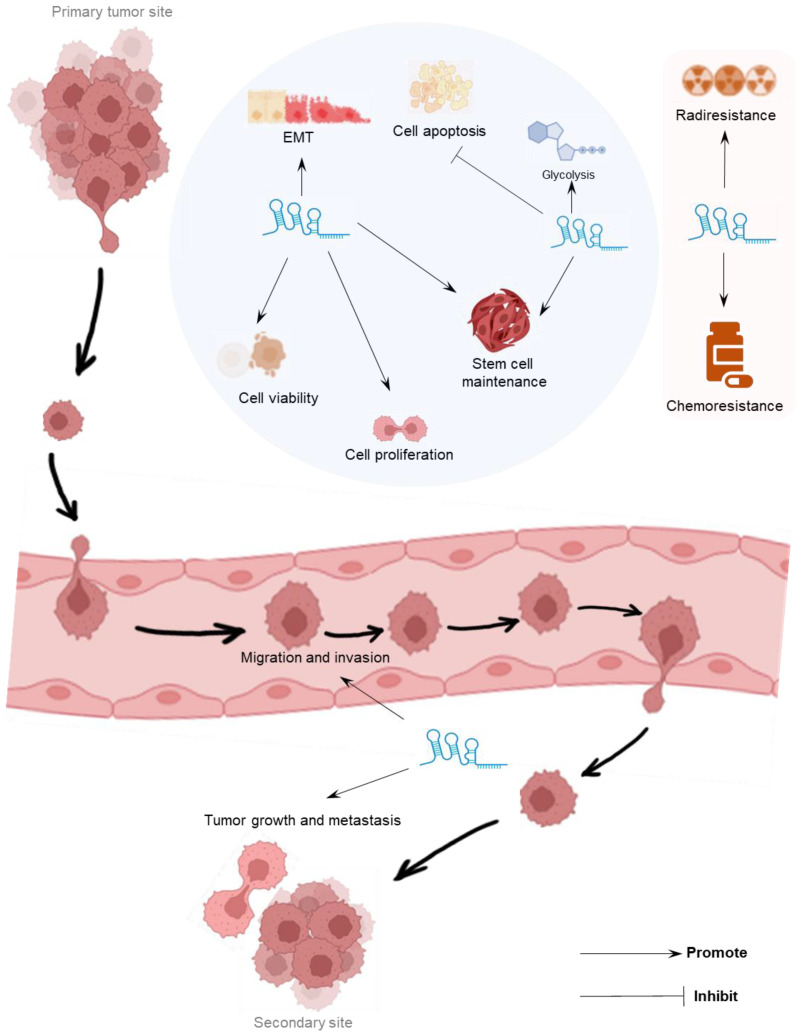
Oncogenic effects of LINC00839 on multiple biological processes leading to cancer progression.

Recent studies, including both *in vitro* and *in vivo* experiments, shows that LINC00839 plays a key oncogenic role in tumor progression (**Table 1**). LINC00839 actively contributes to diverse processes, including cell proliferation, viability, migration, invasion, epithelial-mesenchymal transition (EMT), stemness, glycolysis, resistance to treatment, as well as tumor growth and metastasis (**Figure 2**), highlighting its crucial role in the advancement of tumors. Details on LINC00839's specific functions and mechanisms in different tumors are discussed in subsequent sections.

### Tumors of the head and neck system

#### Nasopharyngeal carcinoma

**Figure 3:**
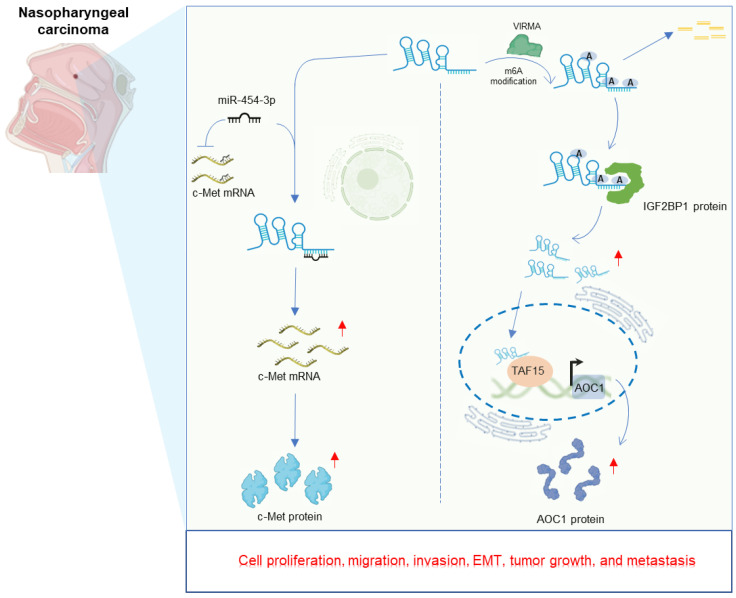
Regulatory mechanisms and consequential effects of LINC00839 in nasopharyngeal carcinoma.

Nasopharyngeal carcinoma (NPC) is a frequent subtype of head and neck cancer, endemic in Southern China, Southeastern Asia and North Africa 55-57. LINC00839 exhibits elevated expression in NPC cells compared to nasopharyngeal epithelial cell lines. Deletion of LINC00839 curbs rapid growth, invasive capabilities, and epithelial-mesenchymal transition (EMT) of NPC cells *in vitro* 32, 33. *In vivo* experiments employing mouse models further demonstrate that LINC00839 knockdown hampers tumor growth 32, 33 and curtails metastasis 33. Mechanistically, LINC00839 plays a significant role in enhancing the aggressive properties of NPC cells by directly acting as a sponge for miR-454-3p, subsequently leading to an increase in c-Met expression 32. Alternatively, LINC00839 could function as an oncogenic m6A-enriched lncRNA, instigating carcinogenesis via a novel oncogenic axis VIRMA/IGF2BP1-LINC00839-TAF15-AOC1 in NPC 33. The detailed regulatory mechanisms of LINC00839 in NPC is displayed in **Figure 3**.

### Tumors of the respiratory system

#### Lung cancer

Lung cancer is a major global health concern and a pressing public health challenge 58, 59. LINC00839 displayed elevated expression levels within lung cancer cell lines (A549, H460). Remarkably, its suppression led to diminished cell viability, reduced migratory and invasive capabilities, accompanied by an increase in cell apoptosis. Mechanistically, LINC00839 acted as a competitive sponge for miR-519d-3p, subsequently resulting in heightened JMJD6 expression. This molecular interplay further contributes to the progression of lung cancer. The constituents of the LINC00839/miR-519d-3p/JMJD6 axis hold potential as promising biomarkers for lung cancer diagnosis and treatment 35.

### Tumors of the digestive system

#### Liver cancer

**Figure 4:**
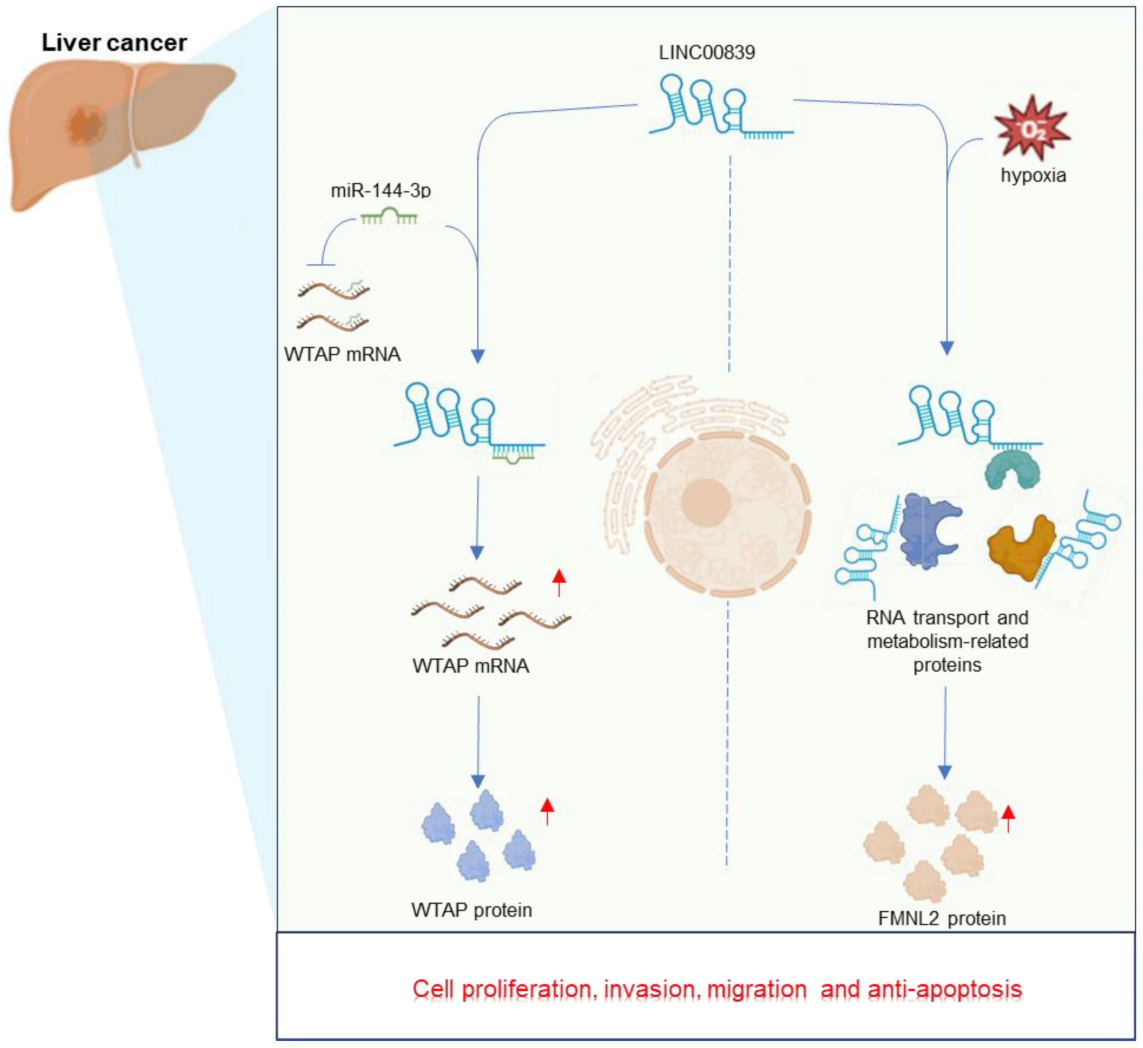
Regulatory mechanisms and multifaceted roles of LINC00839 in the context of liver cancer development.

Liver cancer stands the fourth leading cause of cancer-related deaths worldwide 60. Among primary liver cancers, hepatocellular carcinoma (HCC) accounts for around 90% of cases 61. The increased LINC00839 in liver cancer cell lines, combined with its pivotal oncogenic role in driving HCC development, has been well documented *in vitro* 36, 37. Xie et al. 37 unveiled LINC00839 as a novel hypoxia-responsive long non-coding RNA in liver cancer. Under hypoxic conditions, the overexpression of LINC00839 significantly fueled liver cancer cell proliferation, migration, and invasion. Similarly, another study conducted by Zhou et al. 36 also found that LINC00839 promoted the malignant phenotype of HCC cells. Mechanistically (**Figure 4**), LINC00839 promotes liver cancer progression by interacting with multiple proteins primarily linked to metabolism and RNA transport, and further upregulates FMNL2 expression under hypoxic conditions 37. And LINC00839 can also function as a sponge for miR-144-3p, leading to increased WTAP expression levels 36. Given its significant roles, LINC00839 emerges as a potential therapeutic target and a valuable biomarker for HCC 36, 37. Further *in vivo* studies are essential for future investigations.

#### Gastric cancer

Gastric cancer (GC), a prevalent malignant tumor of the digestive system, still presents challenges in terms of patient prognosis 62. LINC00839 was observed to be upregulated in gastric cancer cell lines (SGC-7901, MGC803, and HGC-27) 38. Functional analyses involving loss and gain assays demonstrated that LINC00839 knockdown inhibits, while LINC00839 overexpression promotes GC cell proliferation, mobility, invasion, and EMT. Further it was confirmed by luciferase assays that LINC00839 acts as a sponge for miR-1236-3p. Notably, miR-1236-3p has been recognized as a tumor suppressor in GC 63. Therefore, LINC00839 might contribute to the progression of GC by sequestering miR-1236-3p 38.

#### Colorectal cancer

Colorectal cancer (CRC) stands as an aggressive malignancy, ranking third in global incidence and second in mortality across all cancers. LINC00839's elevation is evident in CRC cell lines (RKO, HT-29, SW620, HCT116, and CaCo2), alongside its localization within the nucleus 39. Extensive functional investigations underscore LINC00839's role in stimulating CRC proliferation, migration, invasion, and EMT *in vitro*. Moreover, LINC00839's overexpression amplifies tumor growth and metastasis *in vivo*. Mechanistically, LINC00839 orchestrates the recruitment of Ruvb1 to the Tip60 complex, thereby boosting its acetylase activity. This complex, under the guidance of LINC00839, effectively targets the NRF1 promoter, culminating in the acetylation of histones H4 at lysines 5 and 8. Consequently, the expression of NRF1 is enhanced, leading to activation of OXPHOS signaling and mitochondrial metabolism and biogenesis, which collectively drive the progression of CRC 39.

### Tumors of the nervous system

#### Neuroblastoma

**Figure 5:**
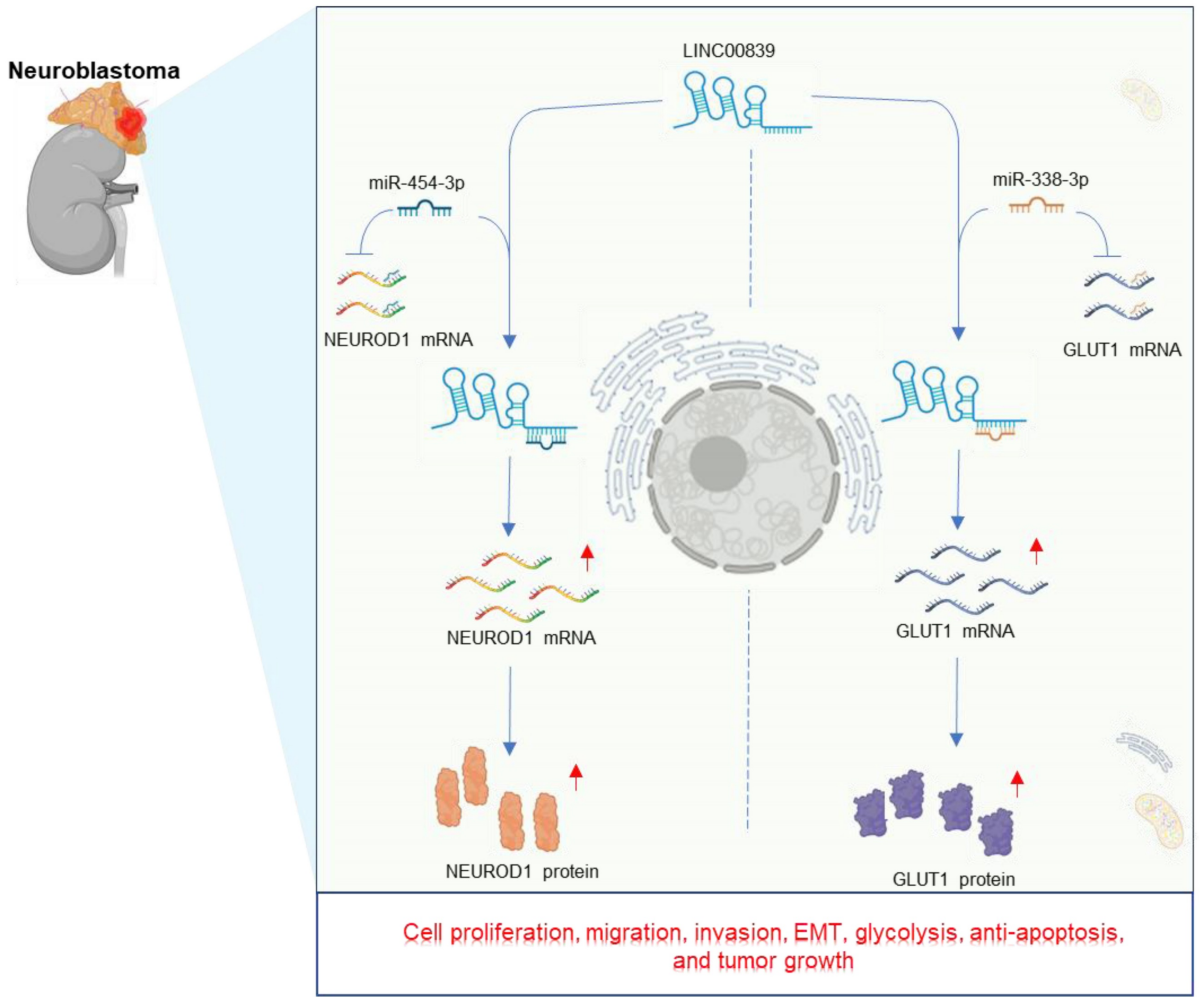
Regulatory mechanisms and effects of LINC00839 in neuroblastoma development. These carcinogenesis effects include cell proliferation, migration, invasion, EMT, glycolysis, anti-apoptosis, and tumor growth.

Neuroblastoma, one of the most common malignant tumors in childhood, is a cancer of the peripheral sympathetic nervous system that is often found in the adrenal gland. LINC00839 levels exhibited an increase in various neuroblastoma cell lines, including SHSY5Y, SK-N-SH, IMR-32, and BE(2)-C. Moreover, its impact on malignant cell phenotypes in neuroblastoma has been investigated 40, 41. Notably, interference with LINC00839 led to a constraint on neuroblastoma cell proliferation, migration, invasion, EMT, and glycolysis, while fostering apoptosis *in vitro* 40, 41. In xenograft mouse models, knockdown of LINC00839 resulted in diminished neuroblastoma growth 40, 41, and also led to extended survival time in mice compared to the control group 40. Mechanistically, LINC00839 plays an oncogenic role in neuroblastoma by upregulating NEUROD1 and GLUT1 expression through sponging miR-454-3p 41 and miR-338-3p 40, respectively. The intricate regulatory pathway of LINC00839 in neuroblastoma is visually depicted in **Figure 5**.

#### Glioblastoma

Glioblastoma, the most common and aggressive form of brain cancer, is marked by symptoms of deteriorating memory, personality, or neurological function, and is pathologically characterized by necrotic tissue surrounded by degenerated cells. Yin et al. 42 uncovered elevated expression of LINC00839 in glioma stem cells (GSCs), contributing to glioblastoma progression and resistance to therapeutic interventions. Through experiments conducted both *in vitro* and *in vivo* 42, LINC00839 emerged as a pivotal factor in nurturing glioma stem cell maintenance and bolstering resistance against radiation. Its overexpression reinforces stemness, while the depletion of LINC00839 elicits contrasting outcomes. In murine models, intracranial xenografts originating from LINC00839 knockdown MES28 cells displayed substantial reductions in tumor volume and extended survival periods. Mechanistically, METTL3-mediated m6A modification influences LINC00839, thereby setting in motion tumor advancement and heightened radiation resistance by activating the Wnt/β-catenin signaling pathway.

### Other systemic tumors

#### Breast cancer

Breast cancer is the most frequently diagnosed malignancy 64, and the second leading cause of cancer-related mortality among females worldwide 65. Chen et al. 34 revealed that Linc00839 localized predominantly within the nucleus and exhibited upregulation in chemo-resistant breast cancer cells. Notably, the knockdown of LINC00839 resulted in a remarkable suppression of proliferation, invasion, and migration, while also fostering cell apoptosis and heightening cellular sensitivity to paclitaxel *in vitro*. These effects extended to inhibiting the development of transplanted tumors *in vivo*. In terms of mechanism, Chen et al. 34 uncovered that Myc could directly bind to the promoter region of LINC00839, thereby instigating its transcription. Moreover, the overexpression of LINC00839 induced heightened expression of Myc and LIN28B, consequently activating the PI3K/AKT signaling pathway. The intricate regulatory network encompassing the Myc/LINC00839/LIN28B feedback loop presents a promising target for innovative therapeutic approaches in breast cancer treatment 34.

#### Bladder cancer

Bladder cancer is a prevalent malignancy in the urinary system 64, 66. Wang et al. 43 uncovered the role of LINC00839 in promoting the spread and drug resistance of bladder cancer cells, specifically against Gemcitabine, a chemotherapy drug. Wang et al. 43 also added a new dimension to this finding by showing EGR1, a transcription factor, directly targets and suppresses LINC00839, leading to a decrease in cancer cell migration and invasion. Furthermore, a complex interaction was discovered where LINC00839 associates with miR-142, thereby influencing the expression of the oncogene SOX5, which is regulated by miR-142. Additionally, EGR1 acts as an inhibitor of SOX5. This regulatory mechanism allows EGR1 to control SOX5 expression both directly and through the LINC00839/miR-142 pathway. A significant aspect of LINC00839's role is its contribution to resistance against Gemcitabine, achieved by enhancing autophagy in cancer cells. Therefore, the EGR1/LINC00839/miR-142/SOX5 loop crucially influences the progression, invasiveness, and chemotherapy resistance of bladder cancer.

## The potential application of LINC00839 in clinical practice

### LINC00839 as a promising predictive marker

The expression levels and clinical significance of LINC00839 have been investigated in multiple studies (**Table 2**). Dysregulation of LINC00839 expression has been observed across various malignancies (**Table 2**). Furthermore, elevated expression of LINC00839 has been significantly associated with unfavorable prognostic outcomes, such as overall survival (OS), disease-free survival (DFS), and advanced clinical features such as lymph node metastasis and advanced clinical stage, in several cancer types (**Table 2**).

**Figure 6:**
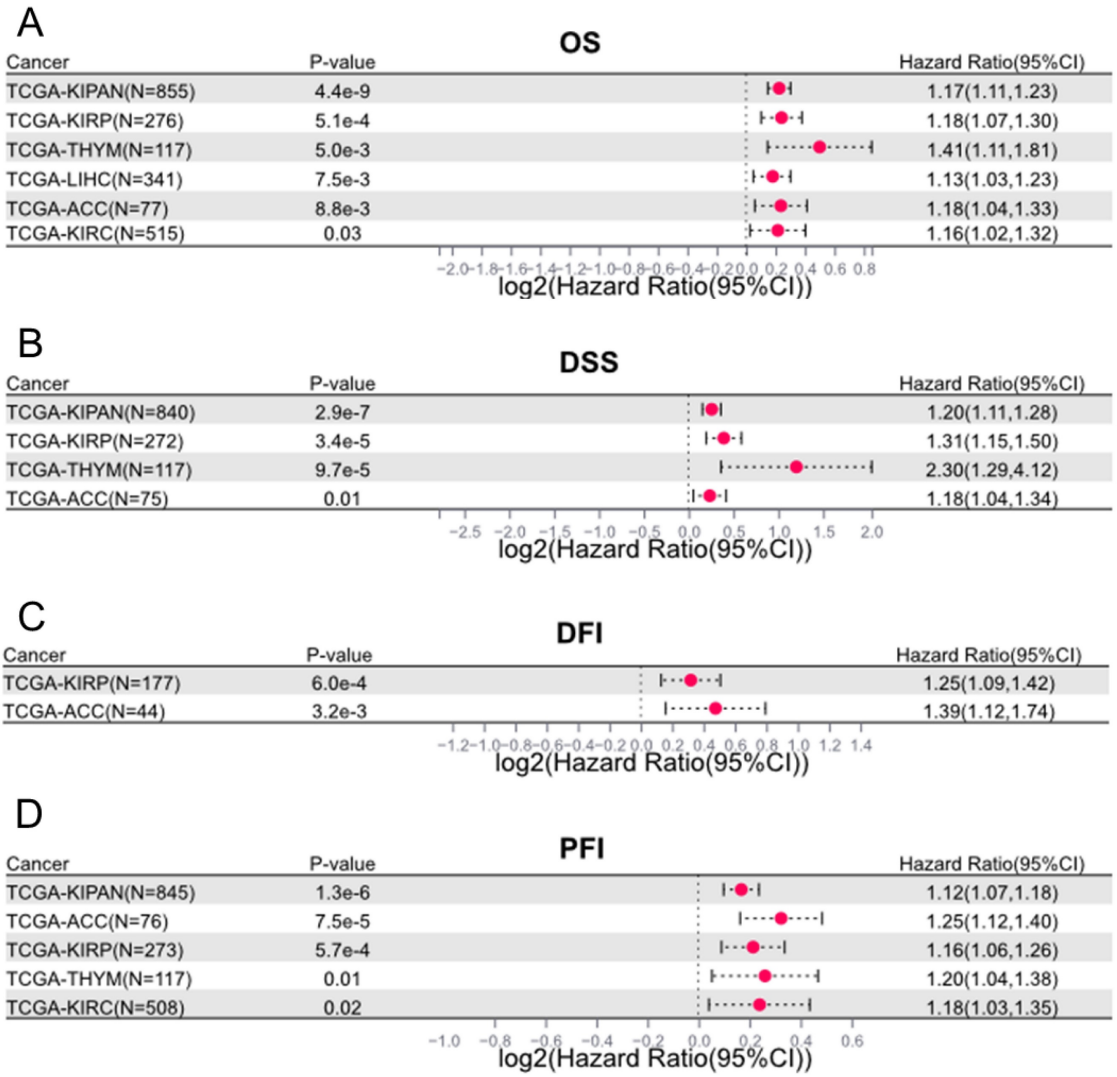
Significant prognostic impact of LINC00839 in additional cancer types beyond those covered in prior publications. The prognostic value of LINC00839 in cancers were assessed in OS (**A**), DSS (**B**), DFI (**C**), and PFI (**D**) using TCGA dataset.

Beyond the cancers examined in previous publications, we also evaluated the prognostic value of LINC00839 in other tumor categories using data from TCGA (https://portal.gdc.cancer.gov/). We found LINC00839 could also serve as a significant prognostic marker in Kidney Renal Papillary Cell Carcinoma (KIRP), Kidney Renal Clear Cell Carcinoma (KIRC), Thymoma (THYM), and Adrenocortical Carcinoma (ACC). In detailed, LINC00839 overexpression was linked to adverse OS and progression-free interval (PFI) in KIRP, KIRC, THYM, and ACC (**Figure 6A and 6D**). Similarly, in disease-specific survival (DSS) analysis (**Figure 6B**), heightened LINC00839 expression was associated with unfavorable outcomes in KIRP, THYM, and ACC. And elevated LINC00839 expression indicated an unfavorable disease-free interval (DFI) in KIRP and ACC (**Figure 6C**).

Notably, the LINC00839-related model may also serve as a valuable tool for assessing the prognosis of cancer patients. A lncRNA signature associated with genomic instability (including BOLA3-AS1, AC004870, and LINC00839) demonstrated strong predictive performance in KIRP 46. And an additional risk signature consisting of LINC00839, LINC01671, AC093673, and AC008760 has been documented to predict the prognosis and immune infiltration of clear-cell renal cell carcinoma 45.

These findings collectively showed the potential of LINC00839 as a robust prognostic biomarker across a range of tumor types. LINC00839 expression might be useful for assessing patient outcomes and tailoring personalized treatment strategies. The correlation between LINC00839 expression and clinical outcomes highlights its clinical utility in guiding therapeutic approaches for cancer patients.

### LINC00839 as a promising therapy target

**Figure 7:**
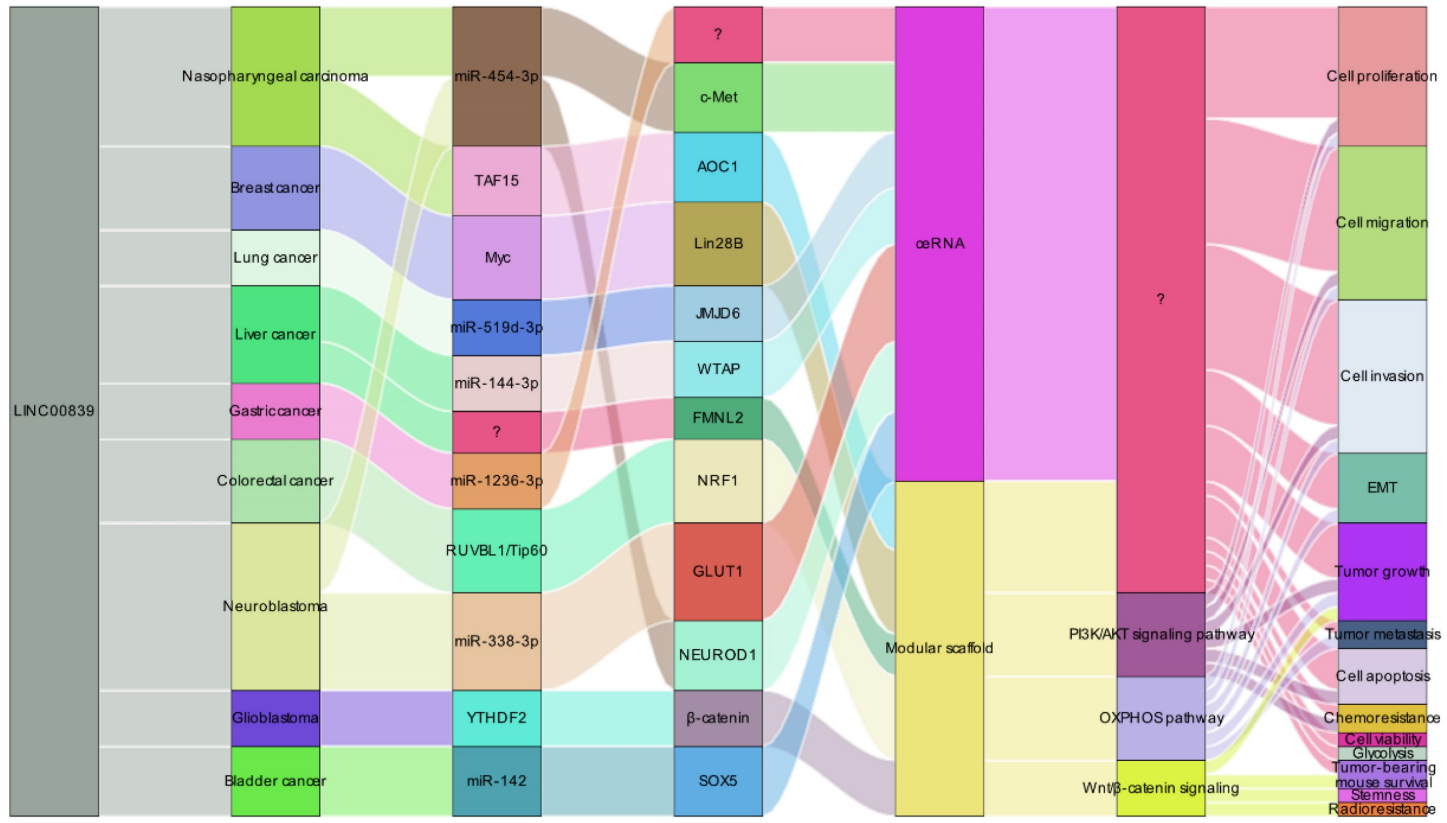
LINC00839's regulatory mechanisms in tumorigenesis and progression. LINC00839 operates as a dual-function regulator, acting as ceRNAs and modular scaffolds. This dynamic role enables LINC00839 to modulate gene expression and impact related signaling pathways, ultimately promoting tumor development.

LINC00839 has garnered attention as a prospective therapeutic target in cancer therapy due to its intricate regulatory capabilities (**Figure 7**). Its remarkable interactions with DNA, protein, and RNA molecules endow it with a role as a versatile modular scaffold, intricately influencing gene expression and shaping cellular dynamics. Functioning as a ceRNA, LINC00839 disrupts microRNA-mediated gene suppression, leading to the upregulation of target genes and a wider regulatory impact. Notably, LINC00839's engagement in pivotal signaling pathways, such as PI3K/AKT, OXPHOS, and Wnt/β-catenin, underscores its central position in maintaining cellular equilibrium and influencing tumor progression. Furthermore, its involvement in critical tumor-related processes including cell migration, invasion, and metastasis accentuates its importance in cancer biology. Impressively, reduced LINC00839 expression has been associated with heightened sensitivity to both drug and radiotherapy treatments, potentially offering a pathway to enhance the effectiveness of existing cancer therapeutic strategies. In sum, LINC00839's intricate network of functions, spanning from regulatory scaffold to its role as a ceRNA and its impact on key signaling pathways, positions it as a promising therapeutic target for innovative cancer interventions aimed at tackling metastasis and refining treatment responses.

## Future perspectives

The emerging landscape of research surrounding the LINC00839 has uncovered its intricate involvement in both non-cancerous and cancerous diseases. In the realm of non-cancerous diseases, LINC00839 has been implicated in acute lung injury 29, osteoarthritis 30, and childhood obesity 31, suggesting its potential as a multifaceted regulator with diverse physiological roles. However, these findings largely stem from predictive analyses and online datasets, warranting further experimental validation to elucidate the underlying mechanisms and therapeutic implications of LINC00839's functions. In acute lung injury, LINC00839 offers a promising avenue for targeted gene therapy. Its ceRNA function in upregulating NLRP3 to promote inflammation and pyroptosis presents LINC00839 as a potential candidate for ALI treatment, with sevoflurane offering a possible route for its modulation. In osteoarthritis, LINC00839's involvement in osteoclast differentiation and extracellular matrix organization necessitates in-depth experimental studies to unveil its precise role in disease progression. Similarly, LINC00839's implications in childhood obesity through the MAPK signaling pathway and apoptosis regulation highlight its potential as a key regulator and a source of new biomarkers and therapeutic targets.

Turning the focus to cancer diseases, LINC00839 demonstrated its carcinogenic ability in a variety of tumor types, including digestive system tumors 36-39, respiratory system tumors 35, nervous system tumors 40-42, and other systemic tumors 32-34. Elevated expression in multiple tumor cell lines and tissues underscores its pivotal role in tumor development. Notably, LINC00839 engages in a myriad of cellular processes including cell proliferation, viability, migration, invasion, EMT, stemness, glycolysis, and therapy resistance. These diverse functions underscore its significance as a critical regulator shaping various facets of tumor progression.

The in-depth exploration of LINC00839's roles across specific tumor types further highlights its clinical relevance. For example, in nasopharyngeal carcinoma 32, 33, LINC00839 acts as a ceRNA to promote aggressive properties, making it a potential therapeutic target. In breast cancer 34, the Myc/Linc00839/Lin28B feedback loop presents an innovative target for therapeutic approaches. For lung cancer 35 and liver cancer 36, 37, LINC00839's interactions with miRNAs and proteins offer potential avenues for biomarker discovery and therapeutic interventions. In colorectal cancer 39, the NRF1-activated OXPHOS pathway, orchestrated by LINC00839, suggests new strategies for targeting tumor metabolism. Moreover, in neuroblastoma 40, 41, LINC00839's modulation of NEUROD1 and GLUT1 expression offers insights into potential therapeutic strategies. As a predictive marker, LINC00839's consistently upregulated expression across various malignancies and its association with adverse prognostic features emphasize its potential as a valuable biomarker. Its diverse roles across different cancer types also highlight its potential as a predictor of disease outcomes. The robustness of LINC00839's predictive value underscores its clinical utility in guiding personalized treatment strategies for cancer patients.

In the realm of cancer therapy, LINC00839's versatile regulatory capabilities and its involvement in crucial signaling pathways position it as an attractive therapeutic target. Its interactions with DNA, proteins, and RNA molecules allow it to act as a regulatory scaffold and a ceRNA, making it a versatile regulator of gene expression. Targeting LINC00839 may offer new avenues for disrupting key pathways, enhancing treatment responses, and curbing metastasis. Its potential to sensitize cancer cells to therapy suggests that manipulating LINC00839 could be harnessed to augment existing therapeutic strategies.

To date, our understanding of the functional implications and clinical significance of LINC00839 in both neoplastic and non-neoplastic conditions is not yet complete. Addressing this requires further exploration of several key aspects. Notably, the specific roles and mechanisms by which LINC00839 functions in non-neoplastic disorders, such as acute lung injury and childhood obesity, are still not fully understood. Moreover, it is important to note that while differential expression of LINC00839 has been reported in certain solid tumors, such as prostate cancer 47, the specific molecular mechanisms involved remain unknown and need to be experimentally investigated *in vivo* and *in vitro*. Additionally, the potential role of LINC00839 in other systemic tumors, especially hematological malignancies, remains completely unexplored and necessitates further study.

Furthermore, although existing studies have demonstrated the diagnostic potential of various long non-coding RNAs 67-73, the diagnostic utility of LINC00839 across diverse disease, including tumors, has not been documented. It is crucial to examine the expression of LINC00839 in pathological tissues and body fluids and its feasibility as a diagnostic marker, and the prognostic and diagnostic value of LINC00839 also needs to be further established in a larger and more diverse population. Moreover, a deeper dive into the intricate mechanisms orchestrated by LINC00839 within different tumors is of paramount importance. LINC00839's involvement might extend to multiple signaling pathways, potentially orchestrating a wider ceRNA network, or acting as a molecular decoy that modulates gene expression. Lastly, a more extensive exploration is imperative to unveil the links between LINC00839 and resistance to therapeutic interventions in more different neoplastic contexts.

## Conclusion

In conclusion, the roles of LINC00839 in both non-cancerous and cancerous diseases have been unveiled through a combination of predictive analyses, experimental studies, and clinical associations. Its diverse functions, ranging from non-cancerous physiological regulation to cancer-related oncogenic processes, underscore its importance in disease progression and therapy. The investigations into LINC00839's mechanisms and therapeutic potential offer a promising outlook for the development of targeted interventions that hold the potential to reshape the landscape of disease treatment. As research continues to unfold, further insights into the intricate functions of LINC00839 are anticipated, paving the way for novel therapeutic strategies and improved patient outcomes.

## Figures and Tables

**Table 1:** The functions and regulatory mechanisms of LINC00839 in various human cancers assessed through tumor cell-based assays and/or mouse model experiments.

Tumor types	Cell lines	Expression in cell lines	Effects *in vitro*	Animal models	Effects *in vivo*	Regulatory mechanism/ Signaling pathway	Ref.
Nasopharyngeal carcinoma	NP-69 and NPC cells (SUNE-1, CNE-2, CNE-1 and C666-1)	Upregulated in cancer cells	proliferation, migration, invasion, EMT	xenograft mouse model	tumor growth	LINC00839/miR-454-3p/c-Met axis	32
	NP69, N2Tert and NPC cells (HONE-1, SUNE-1, C666, HNE-1, 5-8F, 6-10B, S18, S26, CNE-1, CNE-2, and HK-1)	Upregulated in cancer cells	proliferation, migration, invasion	xenograft growth model, lung metastatic colonization model	tumor growth, metastasis	VIRMA/IGF2BP1-LINC00839-TAF15-AOC1 axis	33
Breast cancer	MCF-7, BT549 and MDA-MB-231 cells, MCF-7/ADR cells	Upregulated in MCF-7/ADR cells than in MCF-7, MDA-MB-231, and BT-549 cells	proliferation, migration, invasion, apoptosis, chemoresistance	xenograft mouse model	tumor growth	Myc/LINC00839/Lin28B feedback loop, PI3K/AKT signaling pathway	34
Lung cancer	BEAS-2B and lung cancer cell lines (A549, H460)	Upregulated in cancer cells	cell viability, migration, invasion, apoptosis	/	/	LINC00839/miR-519d-3p /JMJD6 axis	35
Liver cancer	LO2 and HCC cell lines (Huh6, Huh7, SK-hep1, HepG2, and PLC5)	Upregulated in HCC cells	proliferation, invasion, migration, apoptosis	/	/	LINC00839/miR-144-3p /WTAP axis	36
	HL-7702 and liver cancer cell lines (Li-7, SNU-387 and SNU-182)	Upregulated in HCC cells	proliferation, invasion, migration	/	/	Binding FMNL2	37
Gastric cancer	GES-1 and GC cell lines (SGC-7901, MGC803, and HGC-27)	Upregulated in GC cells	proliferation, migration, invasion, EMT	/	/	Sponging miR-1236-3p	38
Colorectal cancer	FHC and CRC cell lines (RKO, HT-29, SW620, HCT116, and CaCo2)	Upregulated in CRC cells	proliferation, migration, invasion, EMT	cell-derived xenograft model, CRC orthotopic mouse model	tumor growth and metastasis	LINC00839-Ruvb1/ Tip60-NRF1, OXPHOS pathway	39
Neuroblastoma	HEK293 and neuroblastoma cell lines (IMR-32 and SK-N-SH)	Markedly elevated in neuroblastoma cells	proliferation, migration, invasion, apoptosis, glycolysis	xenograft model	tumor growth, survival time	LINC00839/miR-338-3p /GLUT1 axis	40
	HEK293 and neuroblastoma cell lines (SHSY5Y, or SK-N-SH, IMR-32 and BE(2)-C)	Markedly elevated in neuroblastoma cells	proliferation, migration, invasion, EMT	subcutaneous xenograft model	tumor growth	LINC00839/miR-454-3p/NEUROD1 axis	41
Glioblastoma	Glioma stem cells GSCs (MES28 and GSC2907) and neural stem cell NSCs (HNP1 and NESA)	Higher expressed in GSCs than NSCs	stemness, radiation resistance	intracranial xenograft model	tumor growth, survival time	YTHDF2/LINC00839/β-catenin, Wnt/β-catenin signaling	42
Bladder cancer	BdEC and bladder cancer cell lines (5637, T24 and J82)	Upregulated in in bladder cancer cells	EMT, migration, invasion, proliferation, chemoresistance	/	/	EGR1/LINC00839/miR-142/SOX5 axis	43

CRC: Colorectal cancer; HCC: Hepatocellular carcinoma; GC: Gastric cancer; GSCs: Glioma stem cells; NSCs: Neural stem cells; EMT: Epithelial-mesenchymal transition; '/' indicates the data is not available.

**Table 2:** Correlation between LINC00839 expression levels in different tumor types, clinical characteristics, and prognosis.

Tumor type	Expression in tumor tissues	Significant clinical features	Prognosis	Ref.
Nasopharyngeal carcinoma	upregulation	/	/	32
Nasopharyngeal carcinoma	upregulation	T-stage, distant metastasis, tumor relapse, locoregional failure, death	OS, DFS, DMFS	33
Breast cancer	upregulation	lymph node metastasis, ER status, TNM stage, Ki-67 level	OS	34
Hepatocellular carcinoma	upregulation	tumor size, lymph node metastasis, clinical stage, tumor differentiation	OS	36
Liver cancer	upregulation	tumor size, number of lesions, pathological grade	OS	37
Gastric cancer	upregulation	/	/	38
Colorectal cancer	upregulation	TNM stage, lymph node status, distant metastasis, vascular invasion, clinical stage	OS, DFS, RFS	39
Neuroblastoma	upregulation	/	OS	40
Neuroblastoma	upregulation	Tumor differentiation, INSS stages, death, MYCN amplification	OS	41
Glioblastoma	upregulation	Tumor recurrent	OS	42
Bladder cancer	upregulation	TNM stages, pathologic stages, histologic grades	/	43
	upregulation	/	/	44
Renal cancer	upregulation	/	OS	46
	upregulation	/	OS	45
Prostate cancer	downregulation	/	/	47

OS: overall survival; RFS: relapse-free survival; DFS: disease-free survival; DMFS: distant metastasis-free survival; ER status: Estrogen Receptor status; TNM stages: Tumor, Nodes, Metastasis stage; INSS stages: International Neuroblastoma Staging System stages. '/' indicates the data is not available.

## Abbreviations

LINC00839: Long Intergenic Non-Protein Coding RNA 839
TCGA: The Cancer Genome Atlas
ALI: Acute lung injury
ceRNA: Competing endogenous RNA
OA: Osteoarthritis
EMT: Epithelial-mesenchymal transition
CRC: Colorectal cancer
HCC: Hepatocellular carcinoma
GC: Gastric cancer
GSCs: Glioma stem cells
NSCs: Neural stem cells
NPC: Nasopharyngeal carcinoma
KIRP: Kidney renal papillary cell carcinoma
KIRC: Kidney renal clear cell carcinoma
THYM: Thymoma
ACC: Adrenocortical carcinoma
OS: Overall survival
PFI: Progression-free interval
DSS: Disease-specific survival
DFI: Disease-free interval
RFS: Relapse-free survival
DMFS: Distant metastasis-free survival
ER status: Estrogen Receptor status
TNM stages: Tumor, Nodes, Metastasis stage
INSS stages: International Neuroblastoma Staging System stages
